# Bacterial profile and antimicrobial resistance patterns of common bacteria among pregnant women with bacteriuria in Ethiopia: a systematic review and meta-analysis

**DOI:** 10.1007/s00404-021-06365-4

**Published:** 2022-01-15

**Authors:** Legese Chelkeba, Korinan Fanta, Temesgen Mulugeta, Tsegaye Melaku

**Affiliations:** 1 Department of Pharmacology and Clinical Pharmacy, School of Pharmacy, College of Health Sciences, Tikur Anbessa Specialized Hospital, Addis Ababa University, Addis Ababa, Ethiopia; 2 Department of Clinical Pharmacy, School of Pharmacy, Institute of Health, Jimma Medical Center, Jimma University, Jimma, Ethiopia

**Keywords:** Antimicrobial resistance, Ethiopia, Pregnant women, Meta-analysis, Significant bacteriuria, Urinary tract infection

## Abstract

**Background:**

Globally, antimicrobial resistance (AMR) restricted the armamentarium of the health care providers against infectious diseases, mainly due to the emergence of multidrug resistant. This review is aimed at providing contemporary bacterial profile and antimicrobial resistance pattern among pregnant women with significant bacteriuria.

**Methods:**

Electronic biomedical databases and indexing services such as PubMed/MEDLINE, Web of Science, EMBASE and Google Scholar were searched. Original records of research articles, available online from 2008 to 2021, addressing the prevalence of significant bacteriuria and AMR pattern among pregnant women and written in English were identified and screened. The relevant data were extracted from included studies using a format prepared in Microsoft Excel and exported to STATA 14.0 software for the outcome measure analyses and subgrouping.

**Results:**

The data of 5894 urine samples from 20 included studies conducted in 8 regions of the country were pooled. The overall pooled estimate of bacteriuria was 15% (95% CI 13–17%, *I*^2^ = 77.94%, *p* < 0.001) with substantial heterogeneity. The pooled estimate of *Escherichia coli* recovered from isolates of 896 urine samples was 41% (95% CI 38–45%) followed by coagulase-negative* Staphylococci*, 22% (95% CI 18–26%), *Staphylococcus aureus*, 15% (95% CI 12–18%), *Staphylococcus saprophytic*, 12% (95% CI 6–18%) *Proteus mirabilis*, 7% (95% CI 4–10%), *Enterococcus* species, 6% (0–12%), *Pseudomonas aeruginosa*, 4% (2–6%), *Citrobacter* species, 4% (95% CI 2–4%), Group B streptococcus, 3% (1–5%), and *Enterobacter* species, 2% (1–4%). Multidrug resistance proportions of *E. coli*, *Klebsiella* species, *Staphylococcus aureus* and *Coagulase negative staphylococci*, 83% (95% CI 76–91%), 78% (95% CI 66–90%), 89% (95% CI 83–96%), and 78% (95% CI 67–88%), respectively.

**Conclusion:**

The result of current review revealed the occurrence of substantial bacteriuria among pregnant women in Ethiopia. Resistance among common bacteria (*E. coli*, *Klebsiella* species, *Staphylococci* species) causing UTIs in pregnant women is widespread to commonly used antibiotics. The high rate of drug resistance in turn warrants the need for regular epidemiological surveillance of antibiotic resistance and implementation of an efficient infection control and stewardship program.

**Supplementary Information:**

The online version contains supplementary material available at 10.1007/s00404-021-06365-4.

## Introduction

Urinary tract infection (UTI) is referred to the invasion of microorganism and their subsequent growth in any part of the urinary tract including the kidneys, ureters, bladder, and urethra [1]. It is one of the most common infectious disease resulting in over 150 million cases per year globally [1, 2]. Although both males and females may become infected by UTI, females are more likely to develop UTI compared to males due to short urethra, proximity to anus which causes easy contamination of the urinary tract with fecal microbial, hormonal changes and pregnancy [3].

The most frequent bacterial infection during pregnancy is urinary tract infection, which is linked to preeclampsia, low birth weight, premature delivery, and intrauterine growth restriction [4]. UTIs are classified as either asymptomatic or symptomatic bacteriuria during pregnancy. The presence of a significant amount of bacteria (10^5^ colony-forming unit (CFU)/ml of cultured urine) in the absence of signs and symptoms of UTI is known as asymptomatic bacteriuria (ABU) [5, 6]. If left untreated, asymptomatic bacteriuria occurs 2–15% of pregnant women and is a common risk factor for pyelonephritis [7, 8]. Although, recent studies question the benefit of ABU treatment particularly, in low-risk pregnant women [7–9], timely initiation of antibiotic as soon as significant bacteriuria is diagnosed likely reduce the incidence of pyelonephritis, preterm delivery, and low birth weight [6]. Several factors are associated with an increased frequency of bacteriuria during pregnancy. These will include age, presence of genitourinary abnormalities (kidney, ureteral and bladder stones, tumors, urethral strictures, vesico-ureteric reflux), anemia, sexual activity, decreased immunity, lower socioeconomic classes, past history of UTI, multiparity and gestational age [10, 11]. For example, the prevalence for bacteriuria increases with age from about 1% in females aged 5–14 years [12].

*Escherichia coli* is the most common pathogenic microorganisms associated with both asymptomatic and symptomatic bacteriuria and accounting up 60–80% of all UTIs in pregnancy [13, 14]. Other bacteria identified in UTIs during pregnancy include *Klebsiella pneumoniae*, *Staphylococcus aureus*,* Coagulase negative staphylococci*, *Enterobacter* spp.,* Pseudomonas aeruginosa*, *Enterococcus* spp.,* Proteus mirabilis*, and others [14].

Antimicrobial resistance (AMR) continued to increase as a result of the rapid emergence of resistant bacteria worldwide [15]. This is demonstrated by recent world health organization (WHO) surveillance data from 22 high- and low-income countries which reported a high level of AMR to numerous bacterial infections and identified *E. coli* and *K. pneumoniae* as the most common resistant pathogen [16]. In addition, meta-analysis of 23 studies from different part of the world reported a high prevalence of extended‐spectrum beta‐lactamase (ESBL)-producing *Enterobacteriaceae* in pregnant women with significant bacteriuria [17]. The resistance of uropathogenic to third-generation cephalosporin, amoxicillin, and other antibiotics in pregnant women especially, in Africa pose a big challenge to the management of UTI during pregnancy [17, 18].

Routine antimicrobial susceptibility test is a major challenge in the majority of low-income countries [19]. As a result, treatment of infectious disease including UTI in pregnant women is initiated empirically and most pregnant women treated with an unnecessary antibiotic which further contributes to the emergence of resistant pathogens [20]. In Ethiopia, there are several studies conducted at different regions and hospitals regarding the bacterial profile and antimicrobial susceptibility patterns of UTI in pregnant women [21–40]. However, there is no pooled prevalence data at the national level which alert health care providers and clinicians on the current prevalence of AMR in UTI during pregnancy. Therefore, this systematic review and meta-analysis is aimed at providing contemporary bacterial profile and antimicrobial resistance pattern among pregnant women with significant bacteriuria.

## Methods

### Reporting

This systematic review and meta-analysis is based on a recommended methodology by the Joanna Briggs Institute’s approach [41] and followed the Preferred Reporting Items for Systematic review and Meta-Analysis for Protocols (PRISMA-P) 2015 guidelines [42].

### Search strategy and data extraction

The present review exhaust all available full-text articles in English language (without date restrictions) indexed in the following major scientific databases: MEDLINE (via PubMed), Web of Science (via Saudi Digital Library), Excerpta Medica dataBASE (EMBASE) and Google scholar. To identify potential search terms and retrieve the best set of results possible, a pre-determined MEDLINE search strategy was constructed using the PICO (patients, interventions, comparators, outcomes) equivalents: patients, exposures of interests, comparison, and outcomes. Accordingly the PECO framework used in our search was described as follows: population (P): pregnant women with bacteriuria in Ethiopia; exposure (E): significant bacteriuria; comparison (C): pregnant women without significant bacteriuria in Ethiopia; outcome (O): bacteria profile, antibiotic resistance profile.

A manual search for additional relevant studies using references from retrieved articles and related systematic reviews was also performed to identify original articles we might have missed. In addition, to find unpublished papers relevant to this systematic review and meta-analysis, some research centers and digital library of higher institutions found in Ethiopia was also explored. The search strings or terms were stemmed from the following keywords*: pregnant women*,* pregnant mother*,* pregnancy*,* bacteriuria*,* urinary tract infection*,* UTI*,* significant bacteriuria*,* uropathogens*,* antibiotic resistance*,* antimicrobial resistance*,* multidrug resistance* and *Ethiopia*. In the advanced searching databases, the searching strategy was built based on the above-mentioned terms using the “Medical Subject Headings (MeSH)” and “All fields” by linking “AND” and “OR” Boolean operator terms as appropriate. The pre-determined MEDLINE search strategy was adopted in searching relevant articles in all other databases. Modifications to indexing terms (e.g., Medical Subject Headings, field tags) for other databases were applied as necessary.

### Data extraction and quality assessment

Data extraction was implemented using a standard and extraction format adapted from the Joanna Briggs Institute (JBI) data extraction format [41]. Screening by title, abstract, and full text and data extraction were done independently by two authors (LC and TM) from the published studies and recorded by Microsoft excel spreadsheet. Consensus was reached by discussion whenever there was disagreement. References and data for each study were carefully cross-checked to ensure that no overlapping data were present and to maintain the integrity of the meta-analysis. Endnote citation manager software version X9 for Windows was utilized to collect and organize search outcomes and for removal of duplicate articles. The retrieved articles were screened according to predefined inclusion and exclusion criteria. We extracted information on name of the first author and year of publication, study period, study design, study region, total sample size, number of isolates, criteria for diagnosis of significant bacteriuria, number and percentage of gram positive and gram negative bacteria, number of asymptomatic and symptomatic bacteriuria and the prevalence and antimicrobial resistance patterns of common bacteria identified. The JBI quality appraisal criteria established for cross-sectional were used to measure the quality of the studies included in the meta-analysis. The JBI’s appraisal criteria consist of nine questions. The first three questions measure about appropriateness of the sampling frame to address the target population, whether participants sampled in appropriate way and sample size is adequate enough. The next three questions measure whether the study subjects and setting of the study described in detail, the data analysis conducted with sufficient coverage of the identified samples and validated for identification method of the condition. The last three questions measure the reliability of measuring the condition in a standard way, the appropriateness of statistical analysis and the adequacy of response rate.

### Inclusion and exclusion criteria

We included published and unpublished (Addis Ababa University) studies reported bacterial profile and their antimicrobial-resistance in pregnant women. We also included studies published in English, human studies and conducted only in Ethiopia. We excluded studies in other population other than in pregnancy, qualitative studies, review paper, commentaries, case series, case reports, conference proceeding and abstracts.

### Data processing and analysis

Data were extracted in Microsoft Excel format, followed by analysis using STATA Version 14.0 statistical software. Random effect model was applied to estimate the pooled estimate and antimicrobial resistance pattern of the isolates. We conducted meta-regression to understand the source of heterogeneity and pooled the estimate using “metaprop” command. Potential source of heterogeneity was investigated by subgroup and meta-regression analysis. The existence of heterogeneity among studies were examined by *I*^2^ heterogeneity test, in which 0–40%, 50–60%, 50–90% and 75–100% represented low, moderate, substantial and considerable heterogeneity, respectively. *I*^2^ heterogeneity test of ≥ 50% and a *p* value of < 0.05 was assured the presence of heterogeneity. Thus, the DerSimonian–Laired random effects model was employed [43]. Subgroup analysis was done based on the patients symptoms status (asymptomatic vs. symptomatic), bacterial isolates (Gram positive vs. Gram negative), geographical region, study period (before 2015 vs. 2015 or after) to minimize the random variations between the point estimates of the primary study. Visual inspection of the funnel plots and Egger’s regression test was used for evaluating the possibility of publication bias. Due to the presence of publication bias the result was corrected by Duval and Tweedie’s trim and fill analysis. Forest plot format was used to present the pooled estimate with 95% CI. Two-sided *p* values < 0.05 were accepted as statistical significant.

### Outcome measurement

This review has three major outcomes. The first outcome was to determine the pooled estimates of significant bacteriuria among pregnant women in Ethiopia. The second outcome was to assess the rate of antimicrobial resistance of common bacteria to common antibiotics used in the country for the treatment of urinary tract infection and finally pattern of multidrug resistance of common uropathogens among pregnant women. The pooled estimates of bacterial isolates and their antimicrobial resistance isolates were calculated using the following formula: a pooled estimate of each isolate was the number of isolate divided by the number of all the detected bacteria isolates. The pooled estimates of resistance to each tested antibiotic was calculated by dividing the number of resistance isolates of each species to total number of all the detected isolates of the species. Multidrug resistance was defined as resistance to at least to two antibiotics to the isolated Gram-positive bacteria for the various antibiotics.

## Results

### Literature identification and characteristics of included studies

**Fig. 1:**
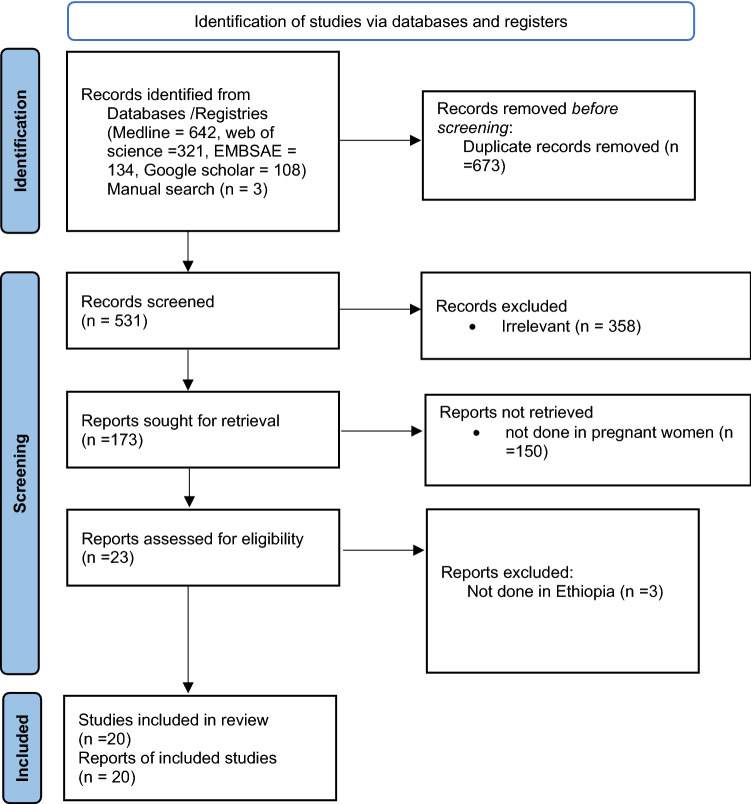
The process of identification, screening, checking for eligibility and inclusion into the final analyses

Of 1204 identified studies (Medline = 642, Web of Science = 321, EMBASE = 134, Google scholar = 108, and manual search = 3), 673 duplicates were excluded. We further excluded 358 studies because they were not relevant up-on reviewing the titles. Then the full-text of the remaining 173 articles was downloaded and fully assessed for fulfilling the required criteria. We again excluded 150 articles because they were not about pregnant women (*N* = 150) and not done in Ethiopia (*N* = 3). Finally, a total of 20 studies were included in this review that met the inclusion criteria [21–40]. Figure 1 summarizes the process of identification, screening, checking for eligibility and inclusion into the final analyses.

All of these studies were conducted from January 2005 to September 2019 and published online from 2008 to 2020. All of the studies were cross sectional in design. All of the studies published on peer review journals except two unpublished data which were obtained from Addis Ababa digital library [44–48]. About one-third of the studies were conducted in Amhara region (*n* = 6) [21, 22, 24, 25, 31, 33] followed by three studies in Addis Ababa city [23, 32, 40], three in Tigray region [26, 37, 38], three in SNNPR [28, 36, 39], two in Oromia region [27, 30], one in Somalia region [29], one in Dire Dawa city [34], and one in Harari region [35]. Sample size of the studies included ranged from 168 [49] to 414 [50]. The prevalence of significant bacteriuria ranged from 7.8 [39] to 24% [34]. Table 1 summarizes the baseline characteristics, tests used, diagnostic criteria, and prevalence of significant bacteriuria among pregnant women.

**Table 1:** Baseline characteristics observational studies included in systematic review and meta-analysis

First author	Year	Region	Study period	Study design	Sample size	Number of isolates	Test	Criteria for diagnosis (NB in ml)	Gram positive *n* (%)	Gram negative *n* (%)	Asymptomatic *n*	Symptomatic *n*	JBI score
1. Biset [30]	2020	Amhara	March to May 2017	Cross sectional	384	61 (15.9%)	Culture	≥ 10^5^ CFU	–	–	23	38	8
2. Tula [36]	2020	SNNPR	March to June 2019	Cross sectional	296	23 (7.8%)	Culture	≥ 10^5^ CFU	5 (22.2%)	18 (77.8%)	12	11	7
3. Derese [31]	2016	Dire Dawa	February 18, 2015 to March 25, 2015	Cross sectional	186	26 (24%)	Culture	≥ 10^5^ CFU	7 (26.9%)	19 (73.1%)	11	15	7
4. Tadesse [33]	2014	SNNPR	March 2012 to September 2012	Cross sectional	244	46 (18.8%)	Culture	≥ 10^5^ CFU	26 (51%)	25 (49%)	46	–	7
5. Tadesse [34]	2018	Tigray	January 1 to April 30, 2018	Cross sectional	259	55 (21.2%)	Culture	≥ 10^5^ CFU	17 (30.9%)	38 (64.1%)	55	–	7
6. Alemu [18]	2012	Amhara	March 22 to April 30, 2011	Cross sectional	385	40 (10.4%)	Culture	≥ 10^5^ CFU	13 (32.5%)	27 (67.5%)	-	–	7
7. Gesesse [24]	2017	Oromia	March 2016 to December, 2016	Cross sectional	300	56 (18.7%)	Culture	≥ 10^5^ CFU	17 (30.4%)	39 (69.6%)	35	21	6
8. Belete [19]	2020	Amhara	February 2017 to May 2017	Cross sectional	323	51 (15.8%)	Culture	≥ 10^5^ CFU	31 (60.8%)	20 (39.2%)	29	21	7
9. Gebremariam [23]	2019	Tigray	February to September 30, 2017	Cross sectional	341	72 (21.1%)	Culture	≥ 10^5^ CFU	27 (36.5%)	47 (63.5%)		72	8
10. Demilie [21]	2012	Amhara	October 2010 to January 2011	Cross sectional	367	35 (9.5%)	Culture	≥ 10^5^ CFU	12 (34.3%)	23 (65.7%)	28	7	6
11 Negussie [26]	2017	Somalia	September 2016 to December 2016	Cross sectional	190	25 (13.2%)	Urinalysis and culture	≥ 10^5^ CFU	6 (24%)	19 (76%)			6
12. Ferede [22]	2012	Amhara	January to May, 2011	Cross sectional	200	24 (12%)	culture	≥ 10^5^ CFU	10 (41.7%)	14 (58.3%)	14	10	6
13. Edae [32]	2020	Harari	March to April 2019	Cross sectional	281	56 (19.9%)	Culture	≥ 10^5^ CFU	22 (39.3%)	34 (60.7%)	56	–	6
14. Wabe [37]	2020	Addis Ababa	July to September 2019	Cross sectional	290	49 (16.9%)	Culture	≥ 10^5^ CFU	20 (40.8%)	29 (59.2%)	49	–	7
15. Tsegaye [35]	2014 (unpublished)	Tigray	January to August 2014	Cross sectional	168	20 (11.9%)	Culture	≥ 10^5^ CFU	8 (40%)	12 (60%)	16	4	7
16. Assefa [29]	2008	Addis Ababa	January to March 2008	Cross sectional	414	50 (11.6%)	Culture	≥ 10^5^ CFU	20 (40%)	30 (60%)	41	9	6
17. Nisha [27]	2015	Oromia	September 2014 to May 2015	Cross sectional	367	59 (16.1%)	Culture	≥ 10^5^ CFU	7	48	59	–	6
18. Bizuwork [20]	2020 (unpublished)	Addis Ababa	March to May, 2019	Cross sectional	281	56 (19.9%)	Culture	≥ 10^5^ CFU	11	45	56	–	7
19. Kumalo [25]	2020	SNNPR	September to December, 2017	Cross sectional	260	27 (10.4%)	Culture	≥ 10^5^ CFU	10	17	27	–	6
20. Ali [28]	2018	Amhara	February to May 2017	Cross sectional	358	56 (15.6%)	Culture	≥ 10^5^ CFU	37	21	56	–	7

*SNNPR* Southern nation, national and people region, *JBI* Joanna Briggs Institute, *CFU* colony-forming unit

### Pooled estimate of significant bacteriuria

**Fig. 2:**
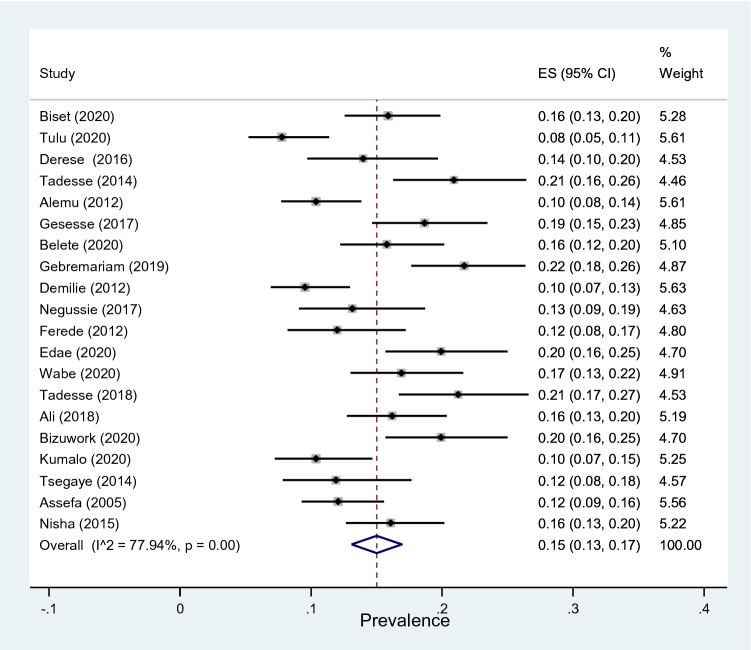
Forest plot showing the overall prevalence of significant bacteriuria among pregnant women in Ethiopia, 2021

A total of 20 studies reported that significant bacteriuria was detected in 896 urine samples out of a total 5894 samples taken from pregnant women during their antenatal care visit to the health care system. In random-effect model, the overall prevalence of significant bacteriuria was 15% (95% CI 13–17%) with substantial heterogeneity (*I*^2^ = 77.94%, *p* < 0.001) across the studies (Fig. 2).

### Subgroup analysis

**Fig. 3:**
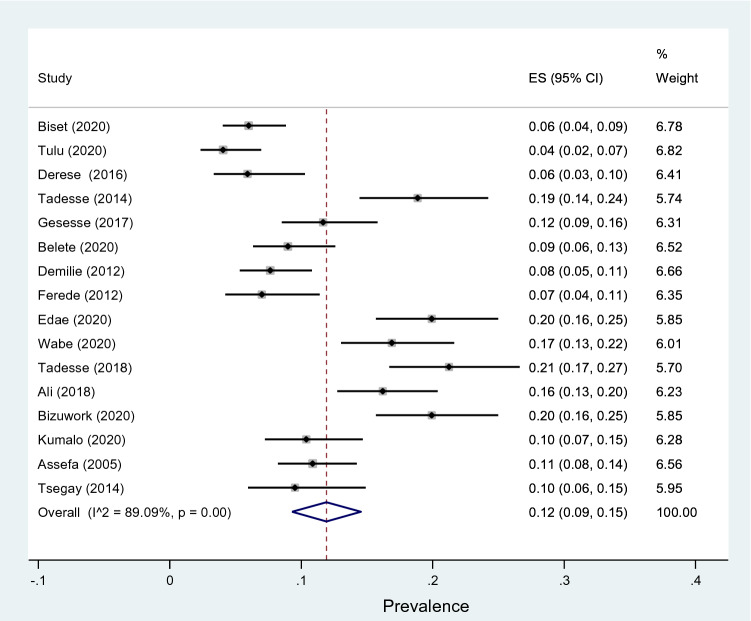
Forest plot showing the prevalence of asymptomatic bacteriuria among pregnant women in Ethiopia, 2021

**Fig. 4:**
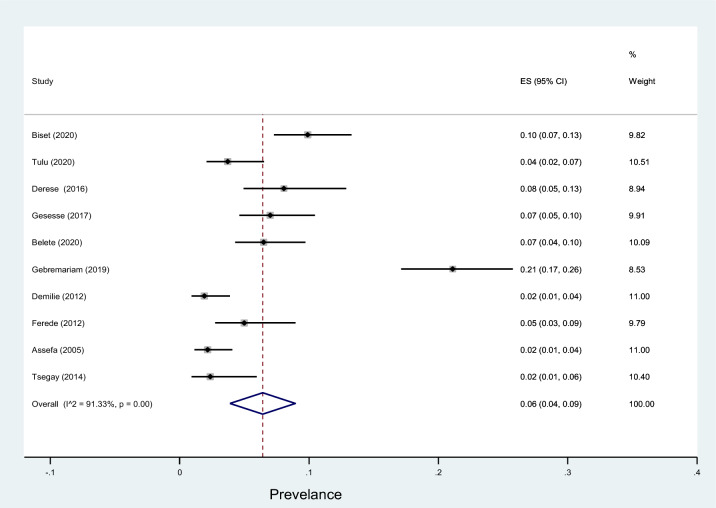
Forest plot showing the prevalence of symptomatic bacteriuria among pregnant women in Ethiopia, 2021

**Fig. 5:**
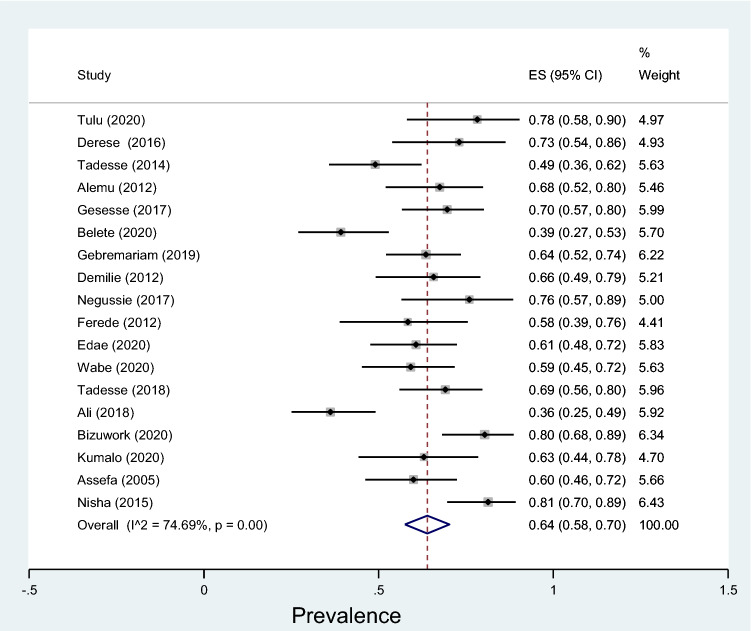
Forest plot showing the prevalence of Gram-negative bacteriuria among pregnant women in Ethiopia, 2021

**Fig. 6:**
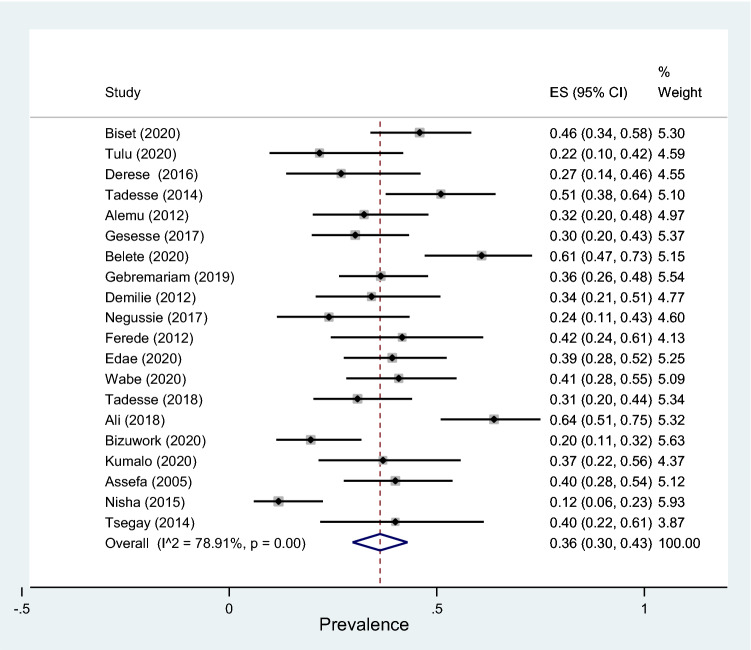
Forest plot showing the prevalence of Gram-positive bacteriuria among pregnant women in Ethiopia, 2021

Subgroup analyses revealed that the proportion of pregnant women developed asymptomatic bacteriuria was 12% (95% CI 9–15%, *I*^2^ = 89.09%, *p* < 0.001) as shown in Fig. 3, whereas only 6% (95% CI 4–9%, *I*^2^ = 91.33%, *p* < 0.001, Fig. 4) developed symptomatic bacteriuria with no statistical significant difference (*p* = 0.14) between the two. Gram-negative bacteria accounted for 64% (95% CI 57–69%, *I*^2^ = 72.80%, *p* < 0.001, Fig. 5) of bacteriuria in participants included in this study, whereas Gram-positive counterparts accounted for 36% (95% CI 30–43%, *I*^2^ = 78.91%, *p* < 0.001, Fig. 6) with a statistically significant difference (*p* = 0.001).

**Fig. 7:**
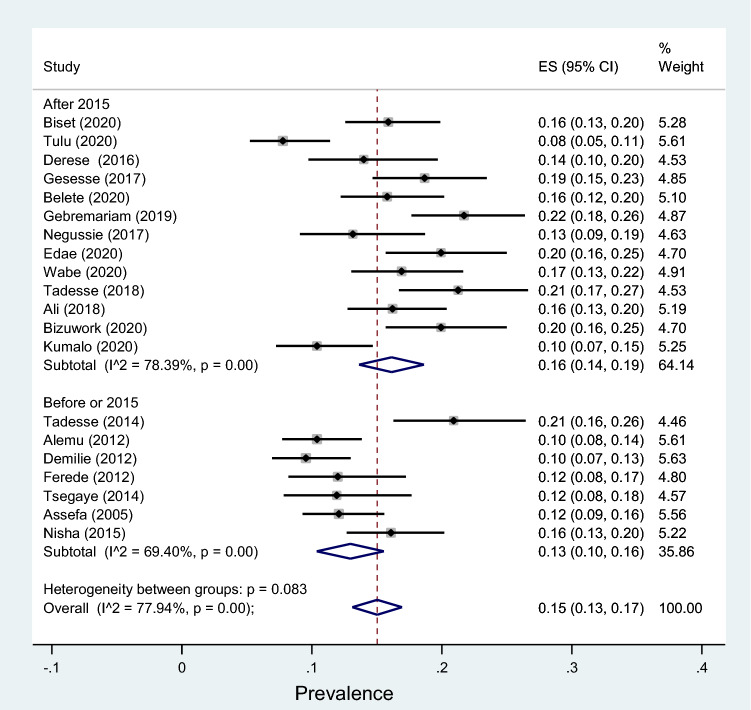
Forest plot showing the prevalence of bacteriuria among pregnant women by study period in Ethiopia, 2021

**Fig. 8:**
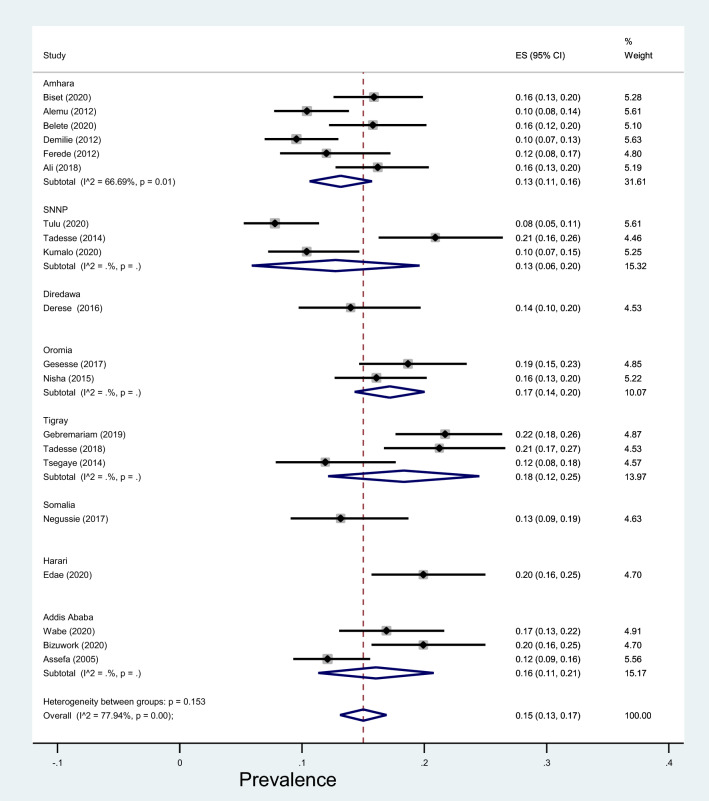
Forest plot showing the prevalence of bacteriuria among pregnant women by region in Ethiopia, 2021

Another subgroup analysis by study period also revealed that the prevalence of significant bacteriuria before 2015 was 13% (95% CI 10–17%, *I*^2^ = 69.40%, *p* < 0.001) while it was 16% (95% CI 14–19%, *I*^2^ = 78.39%, *p* < 0.001) after 2015 with no significant statistical difference (*p* = 0.16) (Fig. 7). Lastly, subgroup by region showed that the prevalence of significant bacteriuria among pregnant women was 16% in Addis Ababa city, 13% in Amhara region, 14% in Dire Dawa city, 20% in Harari region, 17% in Oromia region, 13% in Somalia region, 13% in SNNP region and 18% in Tigray region with no significant difference across the regions (*p* = 0.46) (Fig. 8).

### Meta-regression

Just to identify the sources of heterogeneity, meta-regression was conducted using geographic region, year of publication, and sample size as a covariate. The analysis showed that there is no effect of geographic region (supplementary 1), year of publication (supplementary 2) and sample size (supplementary 3) on heterogeneity between studies (Table 2).

**Table 2:** Meta-regression analysis of some factors assumed to affect between-study heterogeneity

Sources of heterogeneity	Coefficient	Standard error	*p* value
Geographic region	0.020	0.027	0.460
Year of publication	0.027	0.016	0.099
Sample size	− 0.0002	0.001	0.799

### Types of bacterial isolates

**Fig. 9:**
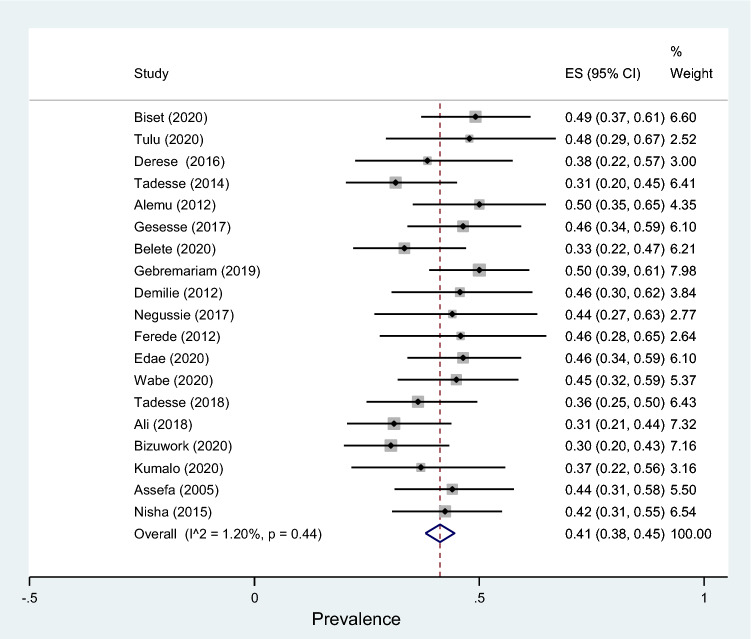
Forest plot showing the prevalence of *E. coli* among pregnant women by region in Ethiopia, 2021

**Fig. 10:**
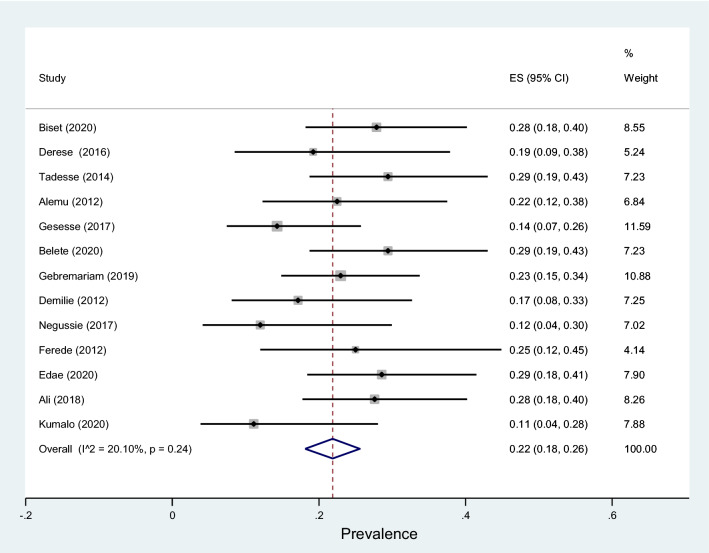
Forest plot showing the prevalence of *Coagulase negative staphylococci* among pregnant women by region in Ethiopia, 2021

**Fig. 11:**
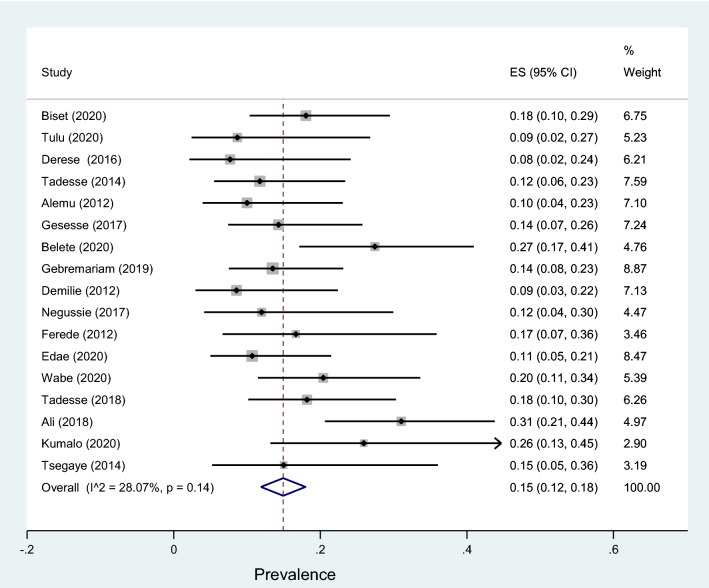
Forest plot showing the prevalence of *S. aureus* among pregnant women by region in Ethiopia, 2021

**Fig. 12:**
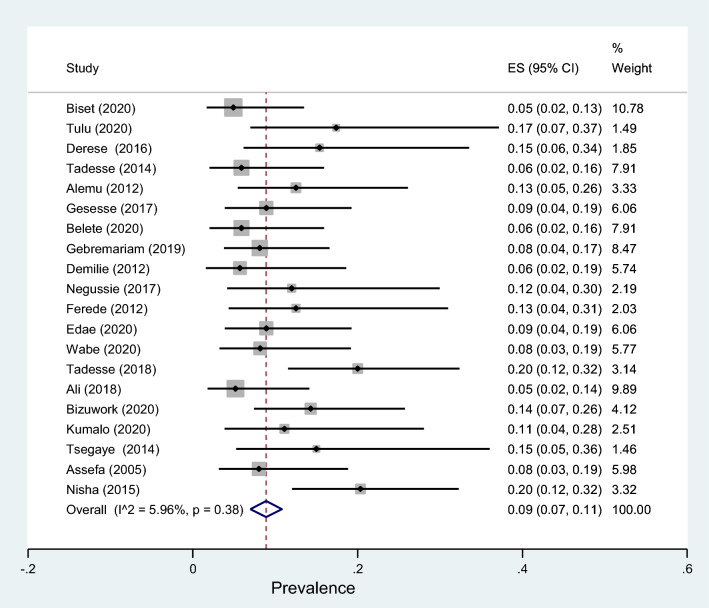
Forest plot showing the prevalence of *Klebsiella* species among pregnant women by region in Ethiopia, 2021

Data of 5894 urine samples from 20 studies conducted in 8 regions of the country were pooled. Eleven different types of bacterial isolates were extracted from studies included in this review. The most common bacterial isolate of both asymptomatic and symptomatic bacteriuria was *E. coli* with an overall prevalence of 41% (95% CI 38–45%, Fig. 9) followed by *Coagulase negative staphylococci* 22 (18–26% Fig. 10), *Staphylococcus aureus* 15% (12–18%, Fig. 11), *Staphylococcus saprophyticus* 12% (7–17%, Supplementary 4), *Klebsiella* species 9% (7–11%, Fig. 12), *Proteus mirabilis* 7% (4–10%, Supplementary 5), *Enterococcus* species 6% (0–12%, Supplementary 6), *P. aeruginosa* 4% (2–6%, Supplementary 7), *Citrobacter* species 4% (2–6%, Supplementary 8), Group B streptococcus 3% (1–5%, Supplementary 9) and *Enterobacter* species 2% (1–4%, Supplementary 10) as depicted on Table 3.

**Table 3:** The estimates of common bacteria isolates of urine samples taken from pregnant women

Bacteria	Number of studies	Sample size	No. of bacteria isolates	Prevalence (95% CI)	*I*^2^ (%)	*p* value
*Escherichia coli*	20	896	371	41 (38–45)	2.03	0.44
*Klebsiella* species	20	896	94	9 (7–11)	5.96	0.38
*Pseudomonas aeruginosa*	10	404	25	4 (2–6)	8.07	0.37
*Proteus mirabilis*	13	560	47	7 (4–10)	52.32	0.02
*Citrobacter* species	10	395	24	4 (2–6)	0.00	0.45
*Enterobacter* species	9	428	16	2 (1–4)	0.00	0.65
*Staphylococcus aureus*	18	781	131	15 (12–18)	00.00	0.14
*Coagulase negative staphylococcus*	15	654	150	22 (18–26)	20.10	0.24
*Staphylococcus saprophyticus*	5	207	29	12 (6–18)	39.51	0.17
*Enterococcus* species	4	180	15	6 (0–12)	69.06	0.02
Group B streptococcus	4	190	7	3 (1–5)	0.00	0.69

### Antibiotic resistance patterns of *E. coli* and *Klebsiella* species isolates

**Fig. 13:**
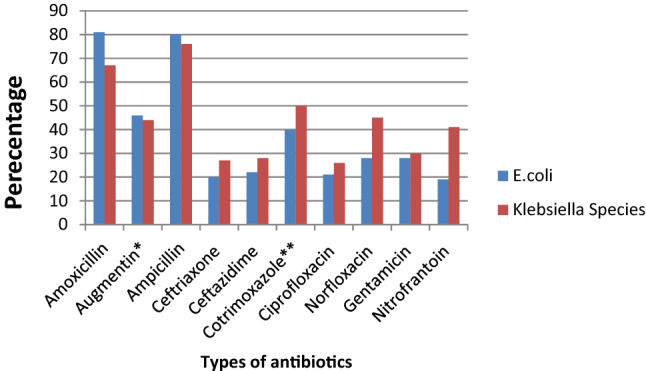
Pattern of antimicrobial resistance of Gram-negative bacteria among pregnant women in Ethiopia. 2021

**Fig. 14:**
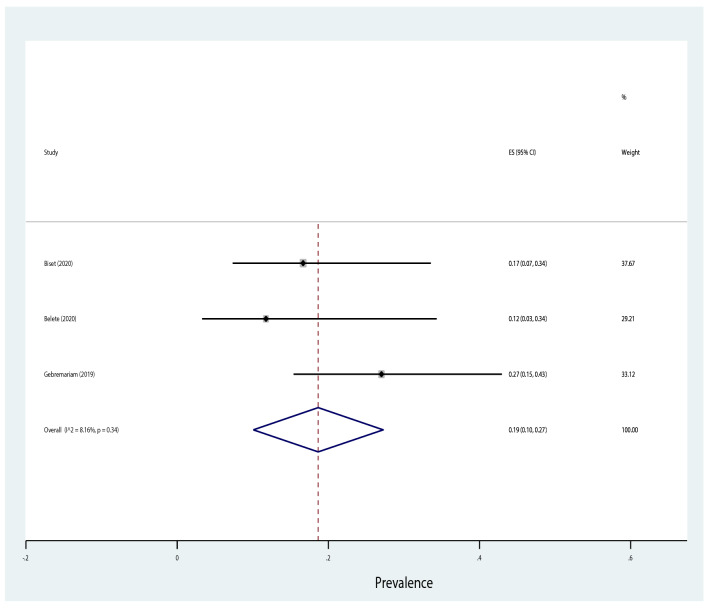
Forest plot showing the prevalence of ESBL *E. coli* among pregnant women

**Fig. 15:**
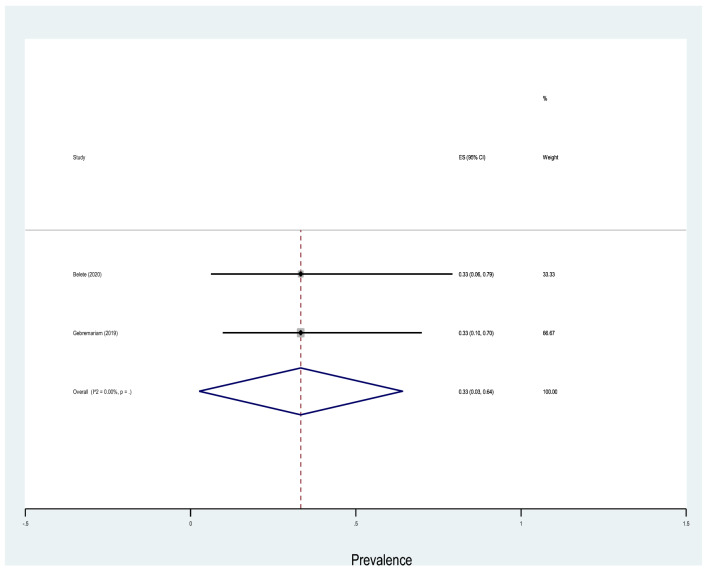
Forest plot showing the prevalence of ESBL *Klebsiella* species among pregnant women

**Fig. 16:**
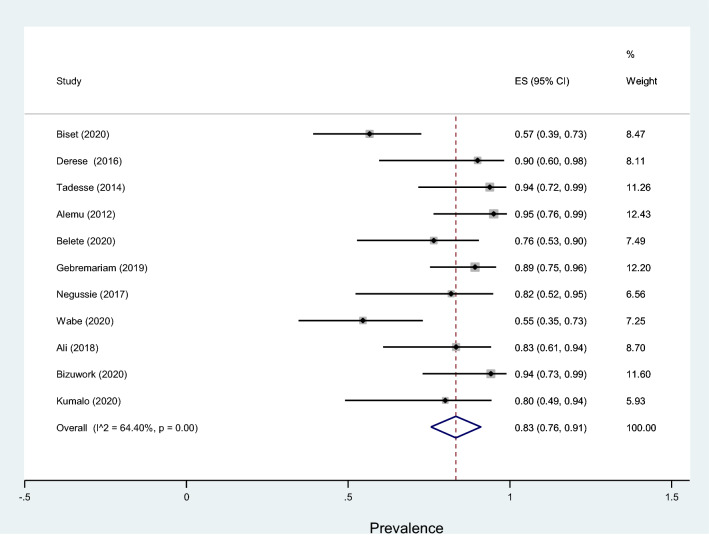
Forest plot showing the prevalence of multidrug-resistant *E. coli* among pregnant women

**Fig. 17:**
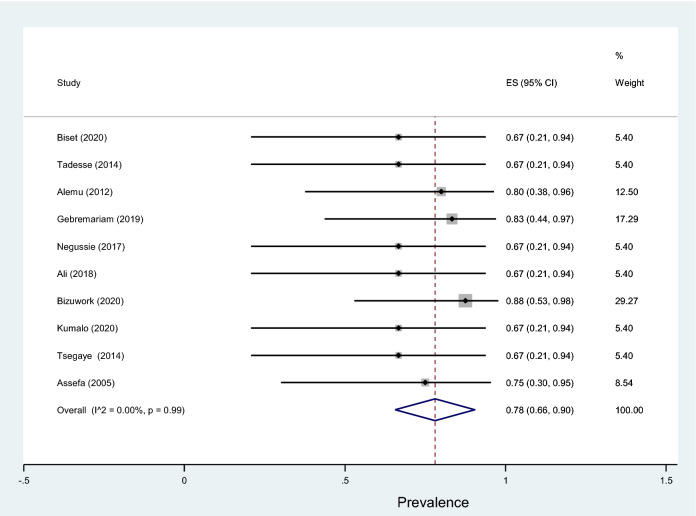
Forest plot showing the prevalence of multidrug-resistant *Klebsiella* species among pregnant women

Table 4 shows that antibiotic resistance pattern for *E. coli* isolated of significant bacteriuria among Ethiopian pregnant women who attained the antenatal care, the most resistance was to amoxicillin with prevalence of 81% (95% CI 69–94%), followed by ampicillin, amoxicillin–clavulanic acid, and sulfamethoxazole–trimethoprim with prevalence of 80% (95% CI 72–88%), 46% (95% CI 29–63%), 40% (95% CI 26–53%), respectively. The lowest resistance was observed to nitrofurantoin with a prevalence of 19% (95% CI 11–27%) followed by ceftriaxone, ciprofloxacin, ceftazidime, gentamicin and norfloxacin with a prevalence of 20%, 21%, 22%, 28% and 28%, respectively. Similarly, the highest rate of resistance by *Klebsiella* species was observed to ampicillin with a prevalence of 76% (95% CI 66–86%) while the lowest resistance was to ceftriaxone with a prevalence of 20% (95% CI 13–28%) as shown in Fig. 13. Three studies reported that the pooled prevalence of ESBL *E. coli* was 19% (95% CI 10–27%, *I*^2^ = 8.16%, *p* = 0.34, Fig. 14), whereas the prevalence of ESBL *Klebsiella* species was 33% (95% CI 3–64%, Fig. 15) reported by two studies. The prevalence of multidrug resistance *E. coli* was 83% (95% CI 76–91%, *I*^2^ = 64.4%, *p* < 0.001, Fig. 16) as reported by 11 studies with a total sample size of 208 and 166 cases while, the prevalence of multidrug-resistant (MDR) *Klebsiella* species was 78% (95% CI 66–90%, *I*^2^ = 00.00%, *p* = 0.99**, **Fig. 17).

**Table 4:** Antimicrobial resistance of *E. coli* and* Klebsiella* species to commonly used antibiotics among pregnant women with significant bacteriuria

Antibiotic	Pooled estimates of resistant isolates									
	*E. coli*					*Klebsiella* species				
	No. of studies	Sample size	Case	ES (95% CI)	*I*^2^ (%)	No. of studies	Sample size	Case	ES (95% CI)	*I*^2^ (%)
Amoxicillin	7	116	90	81 (69–94)	77.42	7	26	17	67 (50–85)	00.00
Augmentin^a^	15	297	140	46 (29–63)	93.12	14	75	33	44 (27–62)	69.72
Ampicillin	17	329	261	80 (72–88)	84.25	15	69	50	76 (66–86)	10.16
Ceftriaxone	13	270	57	20 (13–28)	70.35	15	76	23	27 (16–38)	21.69
Ceftazidime	4	76	17	22 (13–32)	00.00	2	7	2	28 (5–61)	–
Cotrimoxazole^b^	17	135	324	40 (26–53)	89.01	16	80	38	50 (38–62)	17.61
Ciprofloxacin	13	245	45	21 (1–13)	84.35	13	62	18	26 (16–36)	00.00
Norfloxacin	6	104	27	28 (5–50)	91.13	10	38	17	45 (30–60)	00.00
Gentamicin	16	314	90	28 (18–37)	81.26	16	82	27	30 (21–40)	00.00
Nitrofrantoin	9	191	41	19 (11–27)	58.43	8	46	19	41 (27–54)	00.00

^a^Amoxicillin–clavulanic acid

^b^Sulfamethoxazole–trimethoprim

### Antibiotic resistance patterns of *Staphylococcal* species

**Fig. 18:**
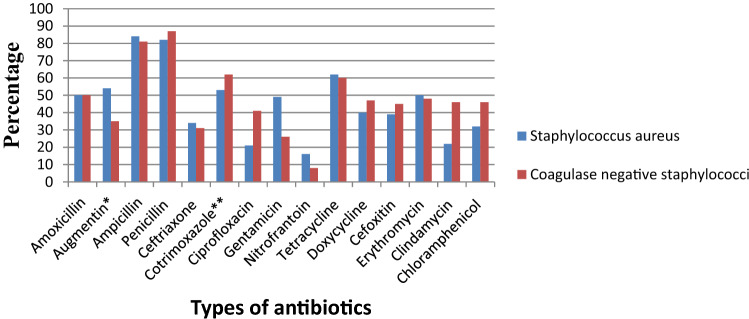
Pattern of antimicrobial resistance of Gram-positive bacteria among pregnant women in Ethiopia, 2021

**Fig. 19:**
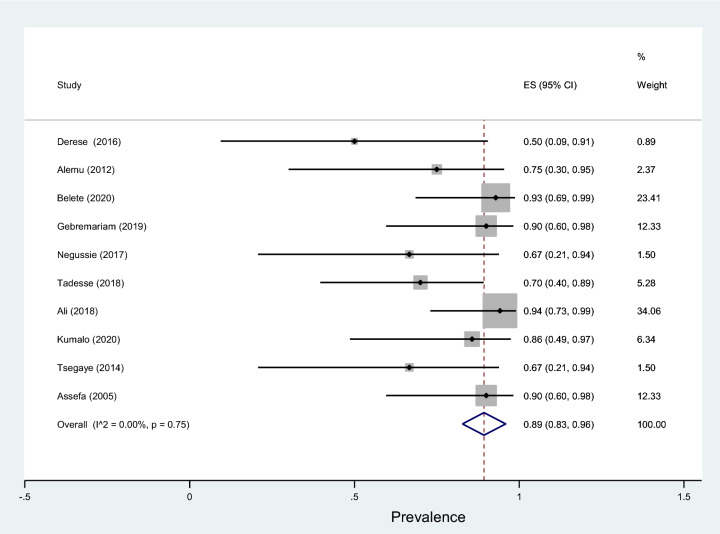
Forest plot showing the prevalence of multidrug-resistant *S. aureus* among pregnant women

**Fig. 20:**
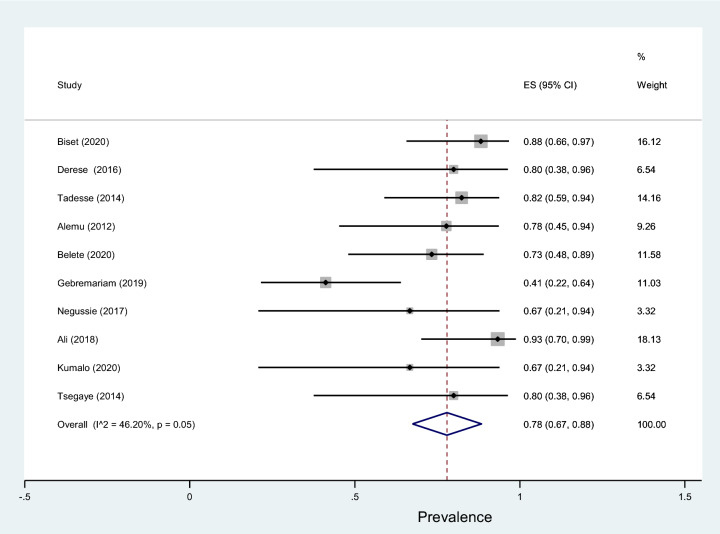
Forest plot showing the prevalence of multidrug-resistant coagulase-negative* Staphylococci* among pregnant women

This review showed that *S. aureus* exhibited the highest resistance to ampicillin, 84% (95% CI 75–93%) followed by penicillin, 82% (95% CI 70–93%), tetracycline, 62% (95% CI 40–88%), amoxicillin–clavulanic acid, 54% (95% CI 10–98%), sulfamethoxazole–trimethoprim, 53% (95% CI 36–70%), and erythromycin, 50% (95% CI 27–73%) as shown in Table 5. Relatively lower rates of resistance were observed to nitrofurantoin, 16% (95% CI 4–49%), ciprofloxacin, 21% (95% CI −98%), clindamycin, 22% (95% CI 11–33%), chloramphenicol, 32% (95% CI 22–42%), and ceftriaxone, 34% (95% CI 18–50%). Similarly, the highest resistance by coagulase-negative* Staphylococci* was observed to penicillin with a prevalence of 87% (95% CI 79–94%) while, the lowest rate of resistance was to nitrofurantoin with a prevalence of 8% (95% CI 0–15%) as depicted in Fig. 18. The prevalence of multidrug resistance isolated *S. aureus* was 89% (95% CI 83–96%, *I*^2^ = 00.00%, *p* = 0.75, Fig. 19) while, it was 78% (95% CI 67–88%, *I*^2^ = 46.20%, *p* = 0.05, Fig. 20) for coagulase-negative *Staphylococci*.

**Table 5:** Antimicrobial resistance of *S. aureus* and coagulase-negative *Staphylococci* to commonly used antibiotics among pregnant women with significant bacteriuria

Antibiotic	Pooled estimates of resistant isolates									
	*S. aureus*					*Coagulase negative staphylococci*				
	No. of studies	Sample size	Case	ES (95% CI)	*I*^2^ (%)	No. of studies	Sample size	Case	ES (95% CI)	*I*^2^ (%)
Amoxicillin	3	10	5	50 (19–81)	00.00	4	26	13	50 (32–69)	00.00
Augmentin^a^	3	25	14	54 (10–98)	86.73	5	40	17	35 (7–64)	79.03
Ampicillin	11	61	49	84 (75–93)	00.00	11	87	68	81 (73–89)	00.00
Penicillin	9	85	68	82 (70–93)	61.08	7	80	67	87 (79–94)	00.00
Ceftriaxone	8	50	18	34 (18–50)	39.46	7	65	22	31 (12–50)	69.49
Cotrimoxazole^b^	16	122	71	53 (36–70)	80.08	13	131	85	62 (45–78)	81.17
Ciprofloxacin	9	67	16	21 (11–30)	00.00	9	86	36	41 (31–51)	00.00
Gentamicin	4	25	12	49 (26–72)	35.92	7	57	18	26 (8–44)	67.92
Nitrofrantoin	6	50	10	16 (4–29)	27.98	4	43	4	8 (0–15)	00.00
Tetracycline	10	78	48	62 (44–80)	71.04	9	95	55	60 (50–69)	00.00
Doxycycline	3	21	8	40 (4–77)	69.91	3	25	13	47 (20–75)	46.33
Cefoxitin	2	18	7	39 (16–61)	–	2	20	9	45 (23–66)	–
Erythromycin	9	86	46	50 (27–73)	84.85	6	77	39	48 (31–64)	58.84
Clindamycin	4	51	12	22 (11–33)	00.00	2	30	14	46 (29–63)	–
Chloramphenicol	10	82	27	32 (22–42)	00.00	10	101	46	46 (31–61)	62.39

^a^Amoxicillin–clavulanic acid

^b^Sulfamethoxazole–trimethoprim

### Publication bias

The asymmetry of funnel plots visual inspection showed that there are some evidence of publication biases in the pooled estimates, which were statistically confirmed by Egger’s regression test (*β* = 8.30, 95% CI 4.30–12.30, *p* < 0.001, Supplementary 11) and after adjusting for publication bias by trim and fill analysis the funnel plot appeared symmetrical (Supplementary 12).

## Discussion

The most prevalent bacterial infection in pregnancy is urinary tract infection (UTI), which increases the risk of maternal and newborn morbidity and mortality [51]. Bacteriuria causes a significantly larger number of neonates with low birth weight, premature delivery, and a higher neonatal mortality rate when it occurs during pregnancy [52]. Thus, this meta-analysis was aimed to determine the burden of bacteriuria among symptomatic and asymptomatic pregnant women, the common bacterial etiologies, and their susceptibility patterns.

In this meta-analysis of 20 studies, 15% of the pregnant women attending the antenatal care follow at different regions of Ethiopia had significant bacteriuria. Our finding showed a slightly higher number of bacteria identified compared to a review study from other low-income countries, where 13.5% of UTI-causing bacterial uropathogens were isolated from the urine sample [14]. In contrast to our meta-analysis, this review included a larger sample size (24,248 vs. 5536 urine specimens), however, the prevalence estimate did not contain the rigorous statistical analysis including the sub-group analysis. However, from a multicentre study of Tanzania, a slightly higher prevalence of significant bacteriuria among pregnant women was reported (17.7%); compared to our finding [19]. In different studies, Greve et al. [53], Lee et al. [54] and Prifti et al. [55], lower findings were reported, where the urine culture positivity (bacteriuria) among 8807, 4034, and 2149 pregnant women were 5.6%, 8.9%, and 13.31%, respectively. Compared with the rest of the world especially the developed countries, the prevalence of bacteriuria among pregnant women in Ethiopia were high. These could be associated with the different modifiable and preventable prenatal factors. These will include anemia, decreased immunity, sexual activity, early age marriage, lower socioeconomic classes, multiparity and gestational age [56–60].

Untreated asymptomatic bacteriuria in pregnancy leads, in as much as 40%, to the development of acute pyelonephritis with all the subsequent negative effects not only for the woman herself but particularly for the foetus [52]. This meta-analytic result indicated 12% of the pregnant women with significant bacteriuria were reported to be asymptomatic. A similar meta-analytic result from Iran indicated 13% of pregnant women had asymptomatic bacteriuria [61]. However, our finding is higher compared to a study by Lee et al. (Bangladesh), where 4.5% of the pregnant women with UTI had asymptomatic bacteriuria [54] and lower compared to a study by Mwambete and Malaba [62] from Tanzania, Dar es Salaam where 23.3% of the pregnant women attending the antenatal care had an asymptomatic urinary tract infection.

In this meta-analysis, Gram-negative bacteria accounted for 64% of culture-positive urine samples. Similarly, a review article from the low-income countries by Belete and Saravanan [14], and a study by Johnson et al. reported Gram-negative bacteria were the most common [63]. Regarding the specific bacteria identified, *E. coli* represented 41% of all the bacteria isolated from 20 studies. Several studies were reported *E. coli* was the most frequent bacteria isolated from the urine culture of pregnant women [19, 53–55, 63–65]. Staphylococcus species were the second frequent uropathogenic bacteria identified after *E. coli*. A similar finding was reported from the community cohort of pregnant women from Bangladesh that staphylococcal species were the second commonest bacterial isolates after *E. coli* [54]. However, a cross-sectional study from Cameroon reported, the Staphylococcal species (45%) were the most frequent bacterial isolates from pregnant women [66]. In contrast, Johnson et al. reported *Klebsiella* species (37.41%) was the most frequently identified bacteria than *E. coli* (28.78%) [63]. The difference might be related to the small sample size of the two studies.

Antibiotic resistance has led to significant challenges in treating UTI [67]. In this meta-analysis, *E. coli* was highly resistant to amoxicillin (81%) and ampicillin (80%). Similarly, Forson et al. from Ghana reported *E. coli* was 79.3% resistant to ampicillin [68]. However, higher resistance of *E. coli* to ampicillin (94.5%) and cotrimoxazole (88.8%) was reported in a multicentre study by Seni et al. compared to our findings. In contrast, in our meta-analysis, lower (40%) of the *E. coli* isolates were resistant to cotrimoxazole [19]. Our findings indicated lower resistance of *E. coli* to the following antibiotics were reported: nitrofurantoin (19%), ceftriaxone (20%), and ciprofloxacin (21%). Similar findings were reported by Seni et al., 12.8%, 13.4%, and 16.5%, respectively [19]. Similarly, in a meta-analysis by Emami et al., *E. coli* was 22% resistant to nitrofurantoin [18]. Our findings showed that 83% of the *E. coli* isolates were MDR and 19% were ESBL producers. Similar findings were reported in another meta-analysis by Mansouri et al. [17] and a study by Sekikubo et al. [20], where 17 and 18% of the *E. coli* strains identified from pregnant women with UTIs were ESBL producers, respectively.

The second frequent Gram-negative isolate in our meta-analysis was *Klebsiella* spp. (9%) from the 20 studies. Similar to *E. coli*,* Klebsiella* spp. were highly resistant to ampicillin (76%). This finding was relatively lower than a study by Seni et al. [19], where about 98% of the *Klebsiella *spp. were resistant to ampicillin. However, lower resistance of *Klebsiella* (21.8%) to ceftriaxone was reported, which is comparable to our finding (20%). Our finding indicated, the pooled prevalence of MDR *Klebsiella* spp. was 78% and 33% of the isolates were ESBL producers. Kaduma et al. reported a lower value; 15.4% of the *Klebsiella* spp. were ESBL producers [69]. The difference might be due to the lower number of participants in a study by Kaduma et al.

In this meta-analysis, *Staphylococcus* spp. isolates were highly resistant to the beta-lactam antibiotics. *S. aureus* was 84% and 82% resistant to ampicillin and penicillin, respectively. And, low resistance to both nitrofurantoin (16%) and ciprofloxacin (21%) identified. A similar resistance profile was noted with coagulase-negative *Staphylococcus*. Similar findings, but a slightly higher percentage of resistance of *S. aureus* to beta-lactam antibiotics was reported by Johnson et al.; ampicillin (90.1%), amoxicillin (93.9%), and amoxicillin/clavulanic acid (78.8%). Similar to our findings, lower resistance to nitrofurantoin (18.2%) and ciprofloxacin (33.3%) was reported [63].

In addition, this meta-analysis result showed the prevalence of multidrug resistance isolated *S. aureus* from urine samples of pregnant women with significant bacteriuria was 89%. Similarly, a study from Nigeria reported *S. aureus* isolated from urine samples of pregnant women was 90.0% each, and 85% resistant to cotrimoxazole, tetracycline, cefoxitin, and vancomycin, respectively [70]. Another study by Asmat et al. [71] also reported all the *S. aureus* isolates were multidrug-resistant; resistant to tetracycline, doxycycline, tobramycin, and intermediately resistant to pipemidic acid. However, a study by Asmat et al. had included a small number of pregnant women.

### Strengths and limitations

The main strength of this meta-analysis is that it was the first to be reported from Ethiopia. In addition, it pooled a relatively large number of articles, which could improve the power and the precision of the estimates of the effect sizes. While the study findings led to the conclusion of high antibiotic resistance in pregnant women with UTI, the original studies included in the analyses came from only six of the country’s nine regions and two cities. As a result, the results’ applicability to the rest of the country may be questioned. In this meta-analysis, there is also significant publication bias, which is graphically assessed using funnel plots and statistically checked for the presence of a small study effect using the Egger test, affecting the interpretation of the findings. However, the pooled prevalence was corrected by Duval and Tweedie’s trim and fill analysis (the same as the original result, 15%). Furthermore, the reader should be aware that the protocol for this study was not made public, which could lead to bias.

## Conclusion

The result of current review revealed the occurrence of substantial bacteriuria among pregnant women in the country. Resistance among common bacteria (*E. coli*, *Klebsiella* species, *Staphylococcus* species) causing UTIs in pregnant women is widespread to commonly used antibiotics. The high rate of bacteriuria among pregnant women in the country is an alert for clinicians to screen bacteriuria at least once using urine culture during antenatal care and treat if urine culture turn around positive. The high rate of drug resistance in turn warrant the need for regular epidemiological surveillance of antibiotic resistance and implementation of an efficient infection control and stewardship program.

## Supplementary Information

Below is the link to the electronic supplementary material.

Supplementary file1 (DOCX 95 KB)

## Data Availability

All relevant data are within the manuscript and its Supporting Information files.
