# Supplementary material for: Bacterial profile and antimicrobial resistance patterns of common bacteria among pregnant women with bacteriuria in Ethiopia: a systematic review and meta-analysis
Source: Arch Gynecol Obstet. 2022 Jan 15;306(3):663–86. doi: 10.1007/s00404-021-06365-4 (PMC9411254; doi:10.1007/s00404-021-06365-4)
Supplement: Supplementary file 1 — Supplementary file1 (DOCX 95 KB) [file 404_2021_6365_MOESM1_ESM.docx]

**Supplementary 1**: Result of meta-regression analysis by region

**Supplementary 2**: Result of meta-regression analysis by study period

**Supplementary 3**: Result of meta-regression analysis by sample size

**Supplementary 4:** Forest plot showing the prevalence of *S. saprophyticus* among pregnant women by region in Ethiopia, 2021

**Supplementary 5:** Forest plot showing the prevalence of *P. mirablis* among pregnant women by region in Ethiopia, 2021

**Supplementary 6:** Forest plot showing the prevalence of *Enterococcus species* among pregnant women by region in Ethiopia, 2021

**Supplementary 7:** Forest plot showing the prevalence of *P.aeruginosa* among pregnant women by region in Ethiopia, 2021

**Supplementary 8:** Forest plot showing the prevalence of Citrobacter species among pregnant women by region in Ethiopia, 2021

**Supplementary 9:** Forest plot showing the prevalence of *Group B streptococcus* among pregnant women by region in Ethiopia, 2021

**Supplementary 10:** Forest plot showing the prevalence of Enterobacter species among pregnant women by region in Ethiopia, 2021

**Supplementary 11**: Funnel pot to test publication bias

**Supplementary 12**: Trim and filled funnel plot after adjusting for publication bias
